# Analysis method of epigenetic DNA methylation to dynamically investigate the functional activity of transcription factors in gene expression

**DOI:** 10.1186/1471-2164-13-532

**Published:** 2012-10-05

**Authors:** Weixing Feng, Zengchao Dong, Bo He, Kejun Wang

**Affiliations:** 1 Pattern Recognition and Intelligent System Institute, Automation College, Harbin Engineering University, Harbin, Heilongjiang, China

**Keywords:** Method, Epigenetics, DNA methylation, Activity, Transcription factor, Gene expression

## Abstract

**Background:**

DNA methylation is a fundamental component of epigenetic modification, which is intimately involved in the regulation of gene expression. One important DNA methylation pathway reduces the abilities of transcription factors to bind to gene promoter regions. Although many experiments have been designed to measure genome-wide DNA methylation levels at high resolution, the meaning of these different DNA methylation levels on transcription factor binding abilities remains poorly understood. We have, therefore, developed a method to quantitatively explore the extent to which DNA methylation levels can significantly reduce or even abolish the binding of certain transcription factors, resulting in reduced or non-expression of flanking genes. This method allows transcription factors that are functionally active in gene expression to be investigated.

**Results:**

The method is based on a general model that depicts the relationship between DNA methylation and transcription factor binding ability based on intrinsic component properties, and the model parameters can be optimized through relative analysis of recognized transcription factor binding status and gene expression profiling. With fixed models, transcription factors functionally active in the regulation of gene expression and affected by epigenetic DNA methylation can be identified and subsequently confirmed. The method identified eleven apparently functionally active transcriptional factors in SH-SY5Y neuroblastoma cells.

**Conclusions:**

Compared with gene regulatory elements, epigenetic modifications are able to change to dynamically respond to signals from physical, biological and social environments. Our proposed method is therefore designed to provide a dynamic assessment to investigate functionally active transcription factors. With the information deduced from our method, we can predict transcription factor binding status in promoter regions to further investigate how a particular gene is regulated by a specific group of transcription factors organized in a particular pattern. This will be helpful in the diagnosis and development of treatment for numerous diseases, including cancer. Although the method only investigates DNA methylation, it has the potential to be applied to more epigenetic factors, such as histone modification.

## Background

Epigenetics is defined as the study of heritable modifications to gene function that occur without alterations in DNA sequences. Epigenetic modifications consist mainly of DNA methylation, histone modifications, chromatin reconstruction, and expression of non-coding RNA. Epigenetic modifications are widely recognized to regulate tissue-specific gene expression, genomic imprinting and X-chromosome inactivation. In addition, the key role of epigenetic modifications during cellular differentiation, development and organogenesis has been highlighted by the identification of many epigenetic biomarkers in human diseases [1,2], such as neuroblastic tumors [3].

The occurrence of many human cancers results from the accumulation of both genetic and epigenetic alterations. While genetic alterations are nearly impossible to reverse, epigenetic alterations can dynamically respond to signals from physical, biological and social environments [4-6]. This characteristic confers the importance of epigenetic research in various cellular processes, especially in gene expression regulation. Although epidemiological data provide evidence that there are direct interactions between epigenetic modifications and the environment to influence gene expression, the mechanism of epigenetic induced modulations of gene expression is still poorly understood.

Regulation of gene expression by transcription factors is a fundamental mechanism. Through the interplay with transcription factors, epigenetic modification such as DNA methylation is able to regulate gene expression [7-11]. For example, high methylation levels in promoter regions always weaken the binding ability of associated transcription factors and cause reduced expression of adjacent genes [12,13]. Although there are many qualitative observations about the effect of DNA methylation on gene regulation, few methods have been developed to assess the effect in a measureable way. Here, we propose a method to evaluate how each transcription factor affects gene expression under a specific pattern of epigenetic DNA methylation levels, which is then used to determine the functional activity of the transcription factor. We describe a general model of how epigenetic DNA methylation affects transcription factor binding ability where several model parameters provide sufficient freedom for different circumstances. Through the relative analysis of recognized transcription factor binding status and gene expression profiling, a model for each transcription factor can be fixed with concrete parameter values. Then, with the deduced models, transcription factors affected by DNA methylation and functionally active in gene expression can be investigated. The proposed method has the capacity to dynamically reflect functions of transcription factors in a temporal and spatial manner.

## Methods

In addition to gene sequence-driven gene regulatory mechanisms, epigenetic modifications, such as DNA methylation, also participate in the regulation of gene expression induced by signals from the environment. Here, based on genome-wide DNA methylation profiling in gene promoter regions, we present a method to investigate transcription factors that are affected by DNA methylation and that are functionally active in gene expression.

### Transcription factor match score

As a functional protein, a transcription factor has the intrinsic tendency to combine with specific DNA sequences, and we define a value termed ‘transcription factor match score’ to evaluate such binding ability for each transcription factor in the promoter region of each gene. In the TRANSFAC database produced by BIOBASE, position weight matrices (PWMs) for every transcription factor are provided. In these matrices, each row consists of four weights representing different capabilities to combine with nucleotides A, C, G and T, respectively. Using these PWMs, each gene promoter region can be scanned nucleotide by nucleotide with a smooth window to compute transcription factor match scores.

For the *ith* transcription factor with a motif length of *L*, a match value at the *kth* putative binding site in promoter region of the *jth* gene can be calculated as *A*_*ijk*_.

(1) $$A_{\text{ijk}}=\sum_{l=1}^{L}a_{\text{jkl}}w_{\text{il}}^{T}$$

where *a*_*jkl*_ is the nucleotide (A, C, G, T) at the *lth* position in the *kth* possible binding site in promoter region of the *jth* gene, and *w*_*il*_ is the *lth* nucleotide in the match weights row vector in the PWM of the *ith* transcription factor. So, suppose the length of the promoter region of the *jth* gene is *N*, *N*-*L*+*1* match values can be calculated and the maximal value is adopted as the match score, *S*_*ij*_, to reflect the binding ability of the *ith* transcription factor in the *jth* gene promoter region.

(2) $$S_{\text{ij}}=\max A_{\text{ijk}}$$

Hence, for one transcription factor, a collection of match scores can be calculated with respect to every gene promoter region.

Although match scores can approximate the opportunity for a transcription factor to bind to a gene promoter region, it is also meaningful to determine a threshold for match scores to evaluate whether the transcription factor binds and regulates the transcription of specific genes.

### Transcription factor match score threshold

As described in the method proposed by Hertzberg [14], for a given transcription factor, a *Z*-score, which considers the relationship between transcription factor match scores and gene expression levels, was calculated to infer the match score threshold.

Suppose there are *n* genes in a cell. Let *g*_*1*_, …,*g*_*n*_ be their log expression values, which would follow normal distribution with an average of *μ* and a standard deviation of *σ*. If a threshold, *t*, is set for match scores of a transcription factor, there will be a subgroup of *k* genes, *G*_*i*_, whose match scores are greater than the threshold *t*, and *G*_*i*_ are assumed to be targets of the transcription factor. The log expression values of these selected genes, *G*_*i*_, also approximately follow normal distribution for a large number of elements in *G*_*i*_. The *Z*-score can then be calculated.

(3) $$Z\left(TF,G^{i}\right)=\left(\frac{1}{k}\sum_{j=1}^{k}g_{\text{ij}}-\mu \right)/\left(\sigma /\sqrt{k}\right)$$

The *Z*-score reflects the extent to which average expression of the selected target genes differ from average expression of all genes. In other words, a larger absolute *Z*-score value means a higher relationship between transcription factor match scores and expression of selected genes, and that these genes are more likely to be regulated by the same transcription factor. Therefore, with different thresholds for transcription factor match scores, we can obtain different groups of transcription factor target genes and subsequently different *Z*-score values. Finally, the best threshold can be determined when the maximal *Z*-score value (if positive) or the minimal *Z*-score value (if negative) is found, where the corresponding *Z*-score for the particular transcription factor is called *Z*_*m*_.

However, without considering the effects of epigenetic modifications, the match score defined above only considers DNA sequences to decide whether a transcription factor binds and regulates the expression of certain genes. This undoubtedly makes subsequent Z-score values inaccurate in the evaluation of transcription factor binding status in gene promoter regions. Hence, we have improved the calculation of the transcription factor match score by adding the effect of epigenetic modifications. However, among the many epigenetic modifications, only DNA methylation was considered because of the requirement for high precision and high resolution data.

### General model of DNA methylation effect

DNA methylation is known to repress transcription factor binding ability [15-23]; therefore, we designed a general model to describe such an effect, where a nonlinear *S*-function is adopted to normalize the effect between 0 and 1. The model consists of two parts. The first sense part uses an inverse *S*-function (Equation 4) to accurately depict the DNA methylation effect. In the equation, *M*_*jk*_ is the methylation level at the *kth* putative transcription factor binding site in the promoter region of the *jth* gene, and *C*_*i*_ and *S*_*i*_ are parameters of the model for the *ith* transcription factor.

(4) $$E_{ijk1}=\frac{e^{-\left(\left(M_{\text{jk}}-C_{i}\right)/S_{i}\right)}}{1+e^{-\left(\left(M_{\text{jk}}-C_{i}\right)/S_{i}\right)}}$$

**Figure 1:**
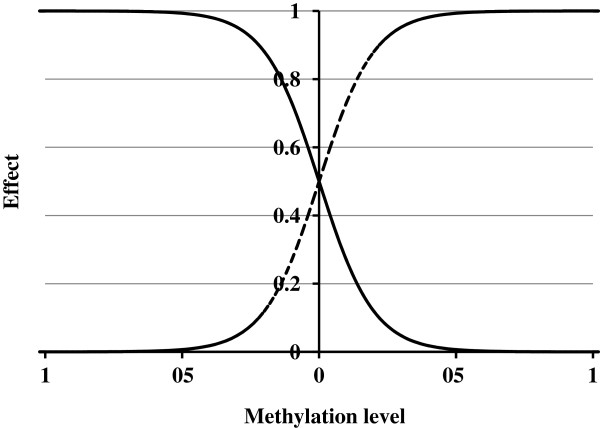
**General model to depict methylation effect (*C*=0, *S*=0.1; solid line: inverse *S*-function, dashed line: normal *S*-function).**

Two biological observations are considered here. A larger methylation level results in reduced transcription factor binding ability in a monotonic way and *vice versa*. Next, the sensitivity of the methylation effect on transcription factor binding ability is not the same at different methylation levels. When the methylation level is quite large or small, the effect tends to be saturating to *0* or *1* and insensitive to a change in methylation level. In contrast, the effect will be sensitive to change when the methylation level is around a median value. Here, an inverse *S*-function is capable of fitting such a relationship, which is shown in Figure 1 (solid line, the parameters are assumed as *C*=*0* and *S*=*0.1*). In Figure 1, the methylation level is on the *X* axis and the suppression of transcription factor binding ability is on the *Y* axis. In the general model, two parameters, *C* and *S*, of the inverse *S*-function are adjustable and can be tuned for different transcription factors in a specific cell.

To increase sensitivity of the method, we also propose a second part to the general model to depict the effect of DNA methylation in an antisense way (Figure 1, dashed line), where a normal *S*-function was used (Equation 5). In the model, a large methylation level was assumed to impact less on transcription factor binding ability and *vice versa*.

(5) $$E_{ijk2}=\frac{1}{1+e^{-\left(\left(M_{\mathrm{jk}}+C_{i}\right)/S_{i}\right)}}$$

### Transcription factor binding score

With consideration of the DNA methylation effect, the binding ability of the *ith* transcription factor in the *jth* gene promoter region can be modified as binding score *B*_*ij*_ from match score *M*_*ij*_.

(6) $$B_{\text{ij}}=\max_{k}\left(A_{\text{ijk}}\times E_{\text{ijk}}\right)$$

where *A*_*ijk*_ is the sequences match value of the *ith* transcription factor and *E*_*ijk*_ is the effect of DNA methylation on the binding ability of the *ith* transcription factor at the *kth* putative binding site in the promoter region of the *jth* gene.

Similar to the transcription factor match score, by threshold analysis of the transcription factor binding score, a maximal *Z*-score value (if positive) or a minimal *Z*-score value (if negative), known as *Z*_*m*_, can also be calculated based on the relative analysis of transcription factor binding scores and gene expression profiles. However, in contrast to only one *Z*_*m*_ value based on the match score, there are many *Z*_*m*_ values for a transcription factor when different compositions of parameters *C* and *S* are selected in the model to calculate different binding scores. Then, when parameters *C* and *S* of the model are fixed to obtain an optimized *Z*_*m*_ value, the effect of methylation on transcription factor binding ability can be quantitatively determined.

### Functionally active transcription factors

According to different ways of describing DNA methylation effects on transcription factor binding ability, three *Z*_*m*_ values can be calculated to investigate functionally active transcription factors. Without considering a DNA methylation effect, *Z*_*m-o*_ is computed when transcription factor match scores are adopted. In contrast, with the consideration of a DNA methylation effect using our proposed model, *Z*_*m-p*_ is analyzed from transcription factor binding scores from the sense orientation and *Z*_*m-q*_ is calculated from transcription factor binding scores from the antisense orientation. Furthermore, with different compositions of model parameters, a group of *Z*_*m-p*_ and *Z*_*m-q*_ values can be calculated for each transcription factor. Then, if absolute *Z*_*m-p*_ values are found to be obviously larger than the absolute *Z*_*m-o*_ value and absolute *Z*_*m-q*_ values are always less than the absolute Z_m-o_ value, the transcription factor is considered to be affected by DNA methylation and functionally active.

## Results and discussion

### Materials

Rett syndrome, a condition frequently seen in cases of developed neuroblastoma, is caused by abnormal interactions between binding proteins and methylated DNA in promoter regions. To evaluate the utility of our proposed method for the investigation of active transcription factors with respect to DNA methylation, a dataset from the SH-SY5Y thrice-cloned neuroblastoma cell line (made by ATCC) was used. As indicated, the dataset includes two parts:

Part 1: DNA methylation levels in promoter regions of SH-SY5Y neuroblastoma cells, assayed with the NimbleGen-1500b-Promoter-Array in the MeDIP experiment, were retrieved (GSE9568). The promoter regions covered by the array are 1200 bps upstream and 300 bps downstream of gene transcriptional start sites. Log2-ratios of the Cy5-labeled test sample versus the Cy3-labeled reference sample were calculated to represent DNA methylation levels. Then, methylation levels of every specific transcription factor binding site (about 10 bps) in all promoter regions were calculated using the Batman algorithm [24].

Part 2: Gene expression levels in the same SH-SY5Y cell line under the same conditions, measured using Affymetrix-HG-U133-plus2.0 GeneChips, were retrieved (GSE4600). Using human RefSeq gene annotations downloaded from the server at UCSC, 10065 available gene expression levels were identified. To enhance observation of the interaction between DNA methylation and transcription factors in the regulation of gene expression, we filtered out genes with very low expression levels.

### Differences in transcription factor binding abilities with and without consideration of a methylation effect

PWMs of 459 human transcription factors were extracted from the TRANSFAC database. With these transcription factor PWMs, match scores for each transcription factor in all gene promoter regions were calculated on human DNA sequences from UCSC. Then, based on DNA methylation levels and gene expression data in SH-SY5Y cells, for each transcription factor we calculated the *Z*_*m-o*_ value, without consideration of the DNA methylation effect, and *Z*_*m-p*_ and *Z*_*m-q*_ values, with consideration of DNA methylation effect, using our proposed sense and antisense models, respectively. Differences among *Z*_*m-o*_ values and extreme *Z*_*m-p*_ values of all transcription factors, with and without consideration of a methylation effect, were found to be significant (Wilcox, P-Value < 2.2e-16), and showed a different distribution of extreme *Z*_*m-p*_ values compared with that of *Z*_*m-o*_ values when considering a DNA methylation effect on transcription factor binding ability.

### Investigation of functionally active transcription factors

Among 459 human transcription factors, E2F1 was reported to be rich in SH-SY5Y cells and to be affected by DNA methylation [25]; therefore, first we show the analysis process of E2F1 in detail.

**Figure 2:**
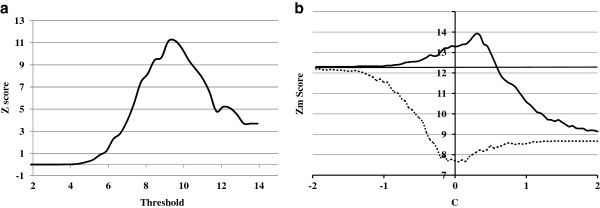
**Analysis of *Z***_***m ***_**scores for E2F1 (a. *Z***_***m-o ***_**value without consideration of methylation effect; b. comparison of *Z***_***m ***_**values with consideration of methylation effect, *S*=0.01, *Z***_***m-o ***_**horizontal line, *Z***_***m-p ***_**solid line, *Z***_***m-q ***_**dashed line).**

Neglecting the effect of methylation, we computed and plotted different Z-scores while adjusting the E2F1 match score threshold (Figure 2a). The positive *Z*_*m*_ values in Figure 2a mean that E2F1 is an active factor in SH-SY5Y cells, which is in accordance with previous studies [25]. The maximal Z-score value (*Z*_*m-o*_) was 12.30 at a match score threshold 9.57.

The effect of methylation with the sense part of model was then considered and *Z*_*m-p*_ values were calculated along with adjustment of model parameters, and the extreme *Z*_*m-p*_ value was found as 13.923 when parameters of the model were selected as *C*=-0.25 and *S*=0.01. The searching process of *Z*_*m-p*_ values is shown in Figure 2b (solid line).

In Figure 2b, the *X* axis is the center *C* of the general model, from -2 to 2 and stepped by 0.05, and the *Y* axis is the corresponding *Z*_*m*_ values (as steepness *S* of the general model contributes little to the effect of the model compared with C, to clearly exhibit the searching process of *Z*_*m*_ values, *S* is set at a fixed value of 0.01). The horizontal solid line at 12.30 indicates the *Z*_*m-o*_ value without consideration of a methylation effect.

In Figure 2b, while increasing the value of *C* from -2 to gradually strengthen the methylation effect, the *Z*_*m-p*_ value begins to rise and soon becomes greater than the *Z*_*m-o*_ value. This means a more reasonable result is obtained when considering a methylation effect on transcription factor binding ability with the sense part of model. The *Z*_*m-p*_ value reaches its highest value at 13.92 (13% higher than the *Z*_*m-o*_ value) when *C* is 0.25. After that, the *Z*_*m-p*_ value drops rapidly when the effect of DNA methylation is further increased.

We also used the antisense part of the model to analyze *Z*_*m-q*_ values along with adjustment of model parameters. The result is shown in Figure 2b (dashed line). While increasing the value of *C* from -2 to gradually weaken the methylation effect, the *Z*_*m-q*_ value reduces and remains lower than the *Z*_*m-o*_ value. This means the antisense part depicts the effect of methylation on E2F1 in an incorrect way. According to our proposed method, we determined that E2F1 was affected by DNA methylation and was functionally active in gene expression in SH-SY5Y cells.

**Figure 3:**
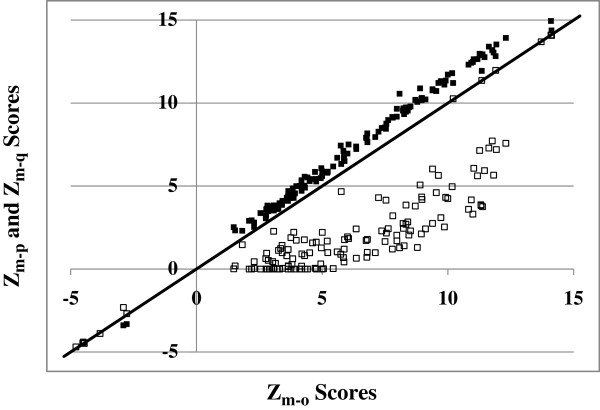
**Comparison of *Z***_***m-o***_**, *Z***_***m-p***_**, *Z***_***m-q ***_**values of active transcription factors (*Z***_***m-p ***_**solid box, *Z***_***m-q ***_**blank box).**

Active transcription factors in SH-SY5Y cells were then investigated. After analysis of distributions of *Z*_*m-o*_, *Z*_*m-p*_ and *Z*_*m-q*_ values of all transcription factors, we adopted two standards to identify active transcription factors. First, an absolute extreme *Z*_*m-p*_ value was at least 10% (90^th^ percentile of ratio values) greater than the absolute *Z*_*m-o*_ value. Second, absolute *Z*_*m-q*_ values were always less than the absolute *Z*_*m-o*_ value. The *Z*_*m-o*_, *Z*_*m-p*_ and *Z*_*m-q*_ values of identified active transcription factors are shown in Figure 3. In Figure 3, the *X* axis is *Z*_*m-o*_ values without consideration of a methylation effect and the *Y* axis is extreme *Z*_*m*_ values with consideration of a methylation effect (extreme *Z*_*m-p*_ values are filled boxes and extreme *Z*_*m-q*_ values are unfilled boxes).

**Figure 4:**
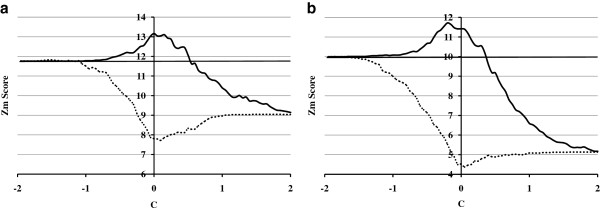
**Comparison of *Z***_***m-o***_**, *Z***_***m-p***_**, *Z***_***m-q ***_**values of listed active transcription factors (*Z***_***m-o ***_**horizontal line, *Z***_***m-p ***_**solid line, *Z***_***m-q ***_**dashed line; a. ZF5; b. AP2).**

Among the identified active transcription factors, the top 10 functionally positive transcription factors (removal of redundant transcription factors) ranked by extreme *Z*_*m-p*_ values in descending order are listed in Table 1. Besides these positive transcription factors, one functionally negative transcription factor, germ cell nuclear factor (GCNF), is listed in Table 2. Comparisons of *Z*_*m-o*_, *Z*_*m-p*_ and *Z*_*m-q*_ values of first two transcription factors (ZF5 and AP2) in Table 1 are shown in Figure 4, and those comparisons of other factors in Tables 1 and 2 are shown in Additional file 1. Similar to E2F1, by calculating and analyzing *Z*_*m*_ values with and without consideration of a methylation effect, the listed transcription factors were found to be in the same pattern as E2F1. Among these active transcription factors, although E2F1, AP2, Sp1 and CREB exist ubiquitously in many tissues, other transcription factors, ZF5, HIF1, AhR and Egr3 were found to specifically exist in SH-SY5Y neuroblastoma cells [26]. Binding of all these transcription factors are found to be affected by DNA methylation [16-21]. Meanwhile, the negative GCNF is also a sequence-specific repressor of transcription through interaction with methylated DNA [22] and functions in neural differentiation [23].

**Table 1:** Top 10 positive transcription factors in SH-SY5Y cells

**No**	**AC**	**Name**	**Z**_**m_o**_	**Extreme Z**_**m-p**_	**C**	**S**
1	M00938	E2F1	12.300	13.923	0.25	0.01
2	M00716	ZF5	11.760	13.189	0	0.01
3	M00189	AP2	9.991	11.714	-0.2	0.1
4	M00196	Sp1	9.908	11.351	-0.05	0.01
5	M00466	HIF1	9.620	11.213	0.15	0.01
6	M00332	Whn	8.892	10.885	0.1	0.01
7	M00778	AhR	9.385	10.768	-0.05	0.01
8	M00801	CREB	8.075	10.562	-0.1	0.05
9	M00245	Egr3	8.965	10.307	-0.1	0.1
10	M00982	KROX	8.701	10.194	0	0.01

**Table 2:** Negative transcription factor in SH-SY5Y cells

**No**	**AC**	**Name**	**Z**_**m_o**_	**Extreme Z**_**m-p**_	**C**	**S**
1	M00526	GCNF	-2.906	-3.390	0.35	0.01

In the method described here, methylation effects on binding abilities of different transcription factors need to be described for each transcription factor; a model is designed with particular independent parameters for each transcription factor. In future research, we will improve the performance of the method by considering transcription factor clustering and multiple transcription factors acting at their binding sites in modules.

## Conclusions

In this study, we have proposed a method to detect active transcription factors in specific cell types by analyzing the interactions between epigenetic methylation patterns in gene promoter regions and the expected binding of transcription factors. In the method, we designed a general model to quantitatively analyze the effect of methylation to suppress transcription factor binding ability in promoter regions, where an inverse *S*-function was adopted to depict the effect of methylation and the model parameters were fixed through calculation of the relationship between transcription factor binding scores in promoter regions and gene expression levels. Based on the model, the case analysis of data from a neuroblastoma cell line successfully showed that 11 transcription factors were obviously affected by methylation of promoter regions and were functionally active in gene expression.

Besides detection of active transcription factors, information deduced from the model can indicate transcription factor binding status in promoter regions to further investigate how a particular gene is regulated by a specific group of transcription factors organized in a particular pattern. This should be helpful for diagnosis and for the development of treatments for numerous diseases, including various cancers. The prediction of transcription factor binding sites produces many false positives; however, by combining static genetic and dynamic epigenetic information together, our proposed approach is capable of effectively decreasing the false positive rate.

So far, we have only considered DNA methylation in the proposed method because of the requirement for high precision and high resolution data. But, the method has the potential to consider more epigenetic factors, such as histone modifications, when the quality of data improves with the development of experimental technology.

## Competing interests

The authors declare that they have no competing interests.

## Authors' contributions

WF and KW designed the study. WF, ZD and BH designed and performed the computational modeling and drafted the manuscript. All the authors read and approved the final manuscript.

## Supplementary Material

Additional file 1 **FigureS1.** Comparison of Zm-o, Zm-p, Zm-q values of Sp1(Zm-o horizontal line, Zm-p solid line, Zm-q dashed line). FigureS2. Comparison of Zm-o, Zm-p, Zm-q values of HIF1(Zm-o horizontal line, Zm-p solid line, Zm-q dashed line). FigureS3. Comparison of Zm-o, Zm-p, Zm-q values of Whn(Zm-o horizontal line, Zm-p solid line, Zm-q dashed line). FigureS4. Comparison of Zm-o, Zm-p, Zm-q values of AhR(Zm-o horizontal line, Zm-p solid line, Zm-q dashed line). FigureS5. Comparison of Zm-o, Zm-p, Zm-q values of CREB(Zm-o horizontal line, Zm-p solid line, Zm-q dashed line). FigureS6. Comparison of Zm-o, Zm-p, Zm-q values of Egr3(Zm-o horizontal line, Zm-p solid line, Zm-q dashed line). FigureS7. Comparison of Zm-o, Zm-p, Zm-q values of KROX(Zm-o horizontal line, Zm-p solid line, Zm-q dashed line). FigureS8. Comparison of Zm-o, Zm-p, Zm-q values of GCNF(Zm-o horizontal line, Zm-p solid line, Zm-q dashed line). Click here for file
