# Supplementary material for: Analysis method of epigenetic DNA methylation to dynamically investigate the functional activity of transcription factors in gene expression
Source: BMC Genomics. 2012 Oct 5;13:532. doi: 10.1186/1471-2164-13-532 (PMC3505177; doi:10.1186/1471-2164-13-532)
Supplement: Additional file 1 — FigureS1. Comparison of Zm-o, Zm-p, Zm-q values of Sp1(Zm-o horizontal line, Zm-p solid line, Zm-q dashed line). FigureS2. Comparison of Zm-o, Zm-p, Zm-q values of HIF1(Zm-o horizontal line, Zm-p solid line, Zm-q dashed line). FigureS3. Comparison of Zm-o, Zm-p, Zm-q values of Whn(Zm-o horizontal line, Zm-p solid line, Zm-q dashed line). FigureS4. Comparison of Zm-o, Zm-p, Zm-q values of AhR(Zm-o horizontal line, Zm-p solid line, Zm-q dashed line). FigureS5. Comparison of Zm-o, Zm-p, Zm-q values of CREB(Zm-o horizontal line, Zm-p solid line, Zm-q dashed line). FigureS6. Comparison of Zm-o, Zm-p, Zm-q values of Egr3(Zm-o horizontal line, Zm-p solid line, Zm-q dashed line). FigureS7. Comparison of Zm-o, Zm-p, Zm-q values of KROX(Zm-o horizontal line, Zm-p solid line, Zm-q dashed line). FigureS8. Comparison of Zm-o, Zm-p, Zm-q values of GCNF(Zm-o horizontal line, Zm-p solid line, Zm-q dashed line). [file 1471-2164-13-532-S1.pdf]

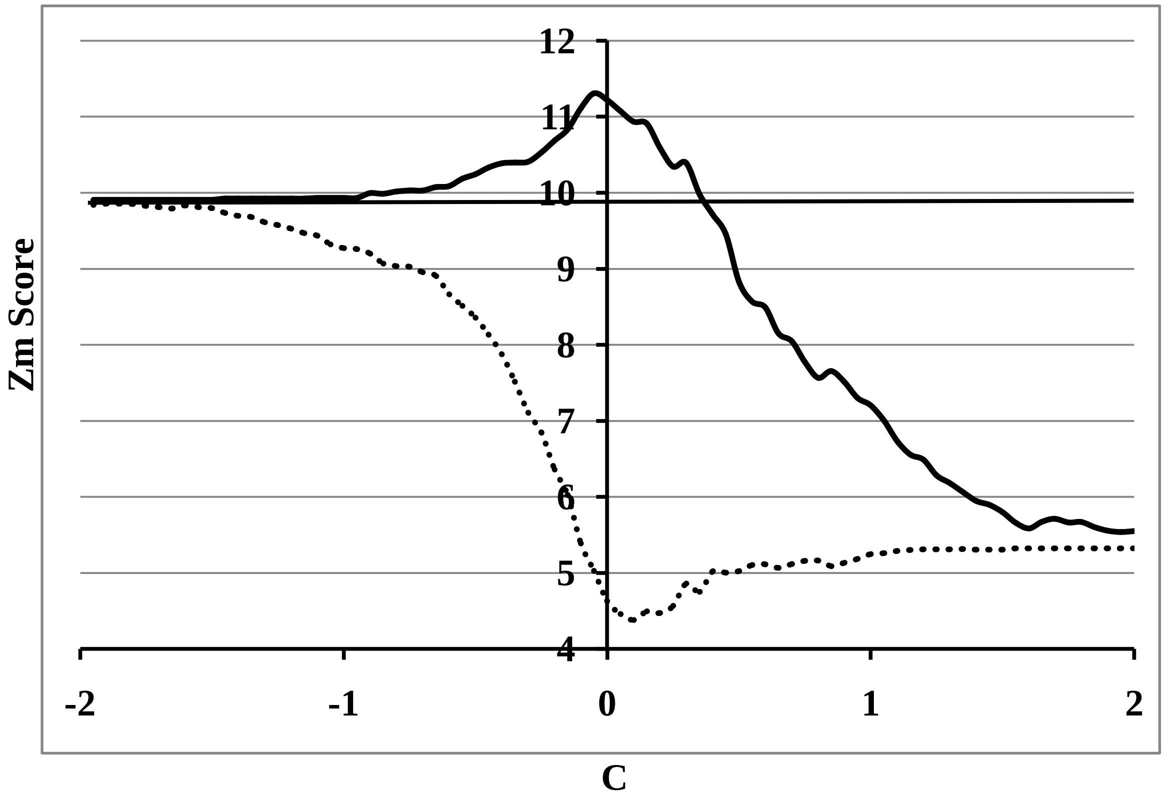

Figure1 Comparison of Zm-o, Zm-p, Zm-q values of Sp1(Zm-o horizontal line, Zm-p solid line, Zm-q dashed line)

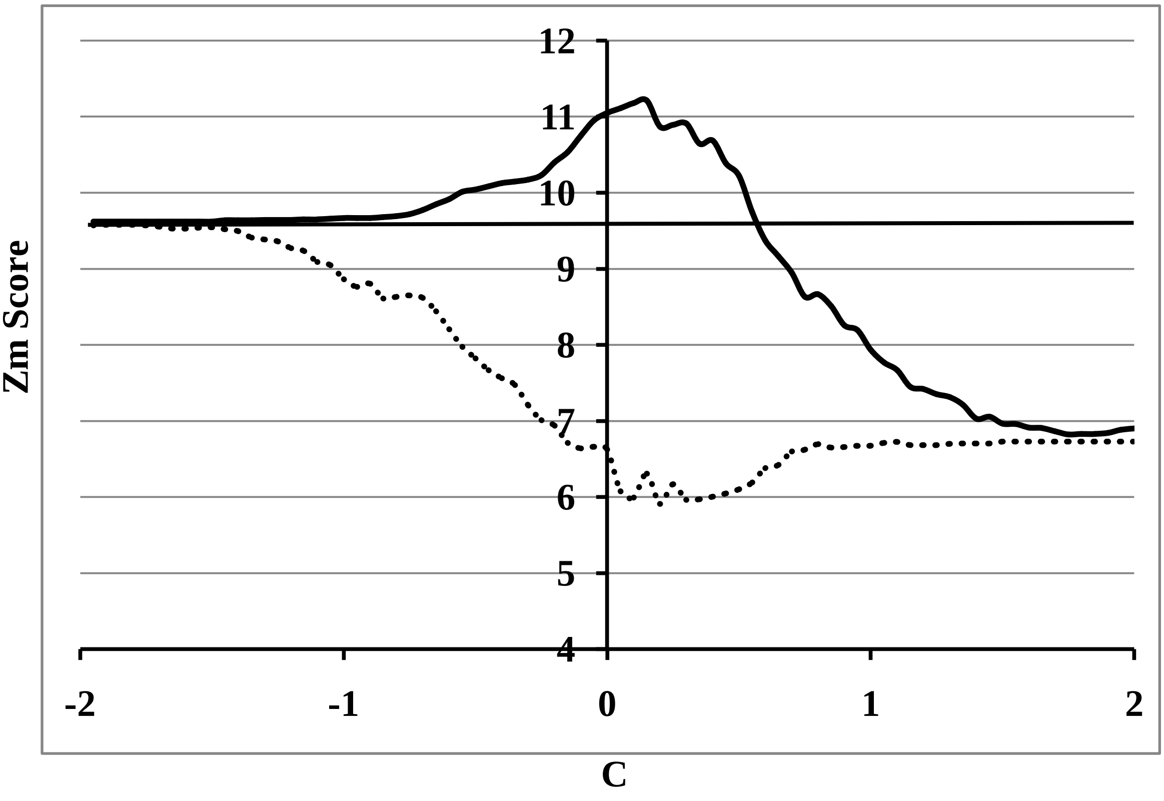

Figure2 Comparison of Zm-o, Zm-p, Zm-q values of HIF1(Zm-o horizontal line, Zm-p solid line, Zm-q dashed line)

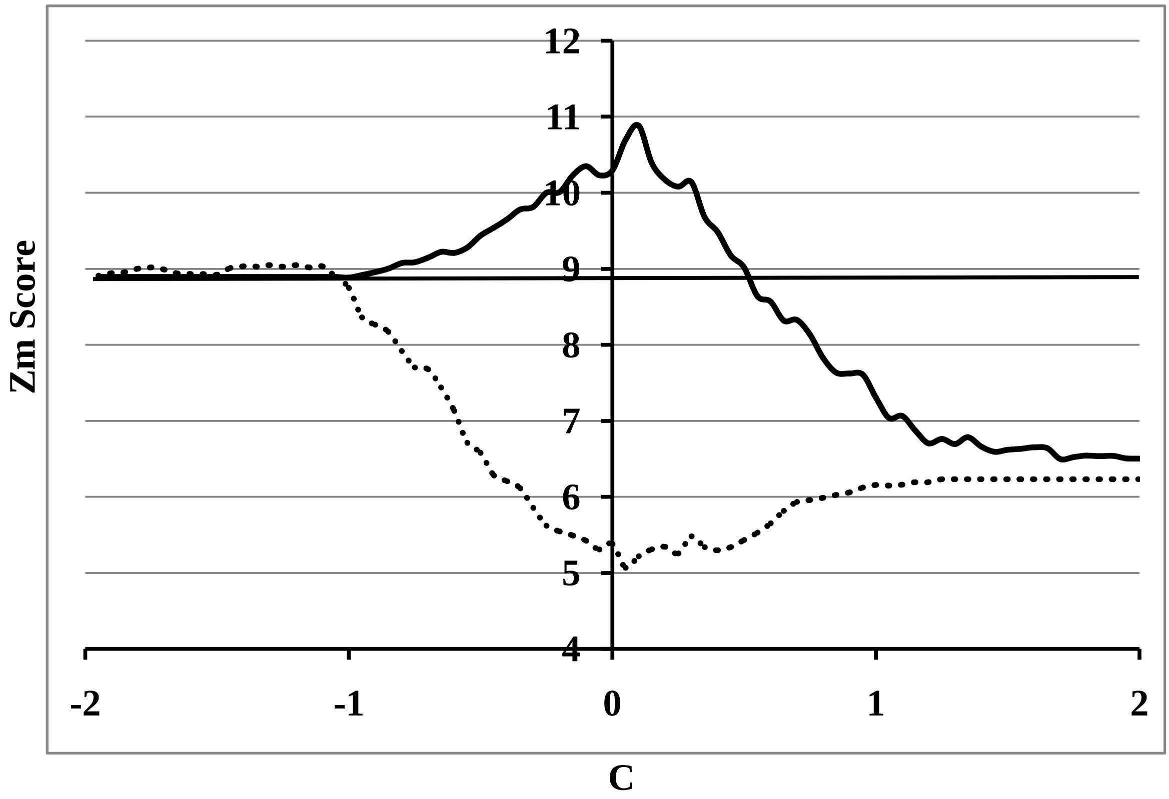

Figure3 Comparison of Zm-o, Zm-p, Zm-q values of Whn(Zm-o horizontal line, Zm-p solid line, Zm-q dashed line)

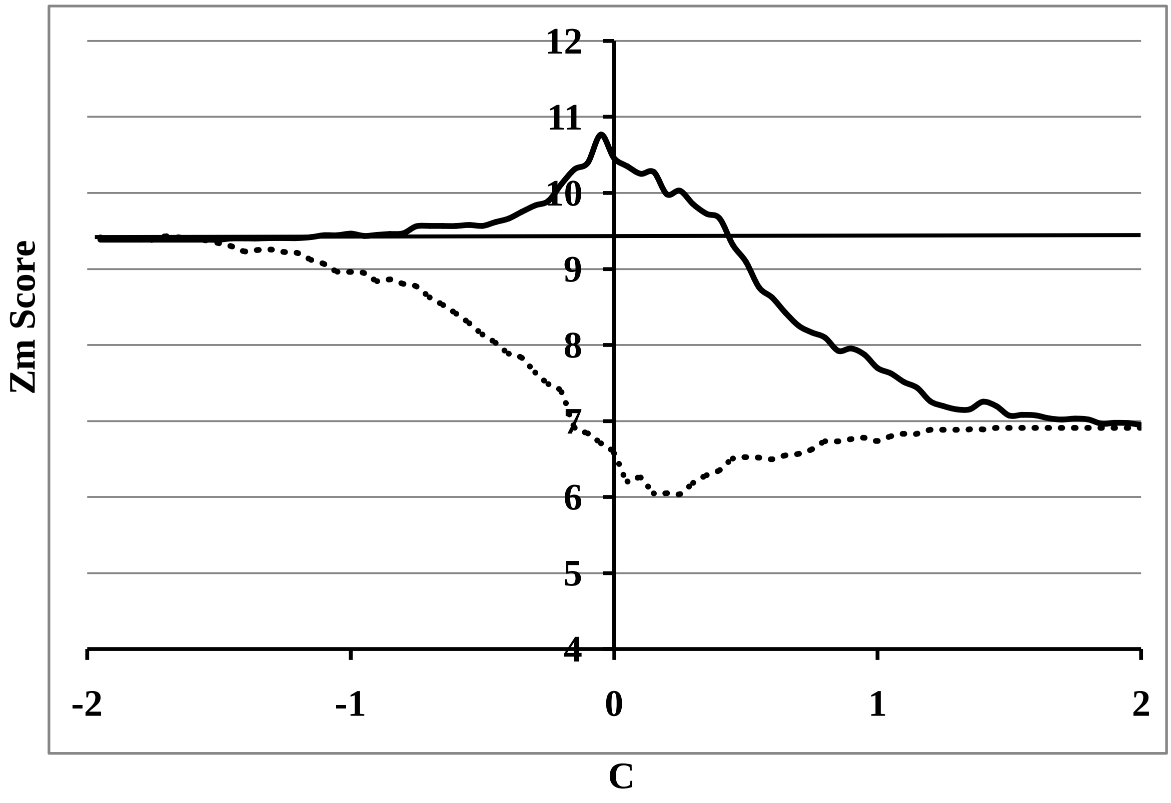

Figure4 Comparison of Zm-o, Zm-p, Zm-q values of AhR(Zm-o horizontal line, Zm-p solid line, Zm-q dashed line)

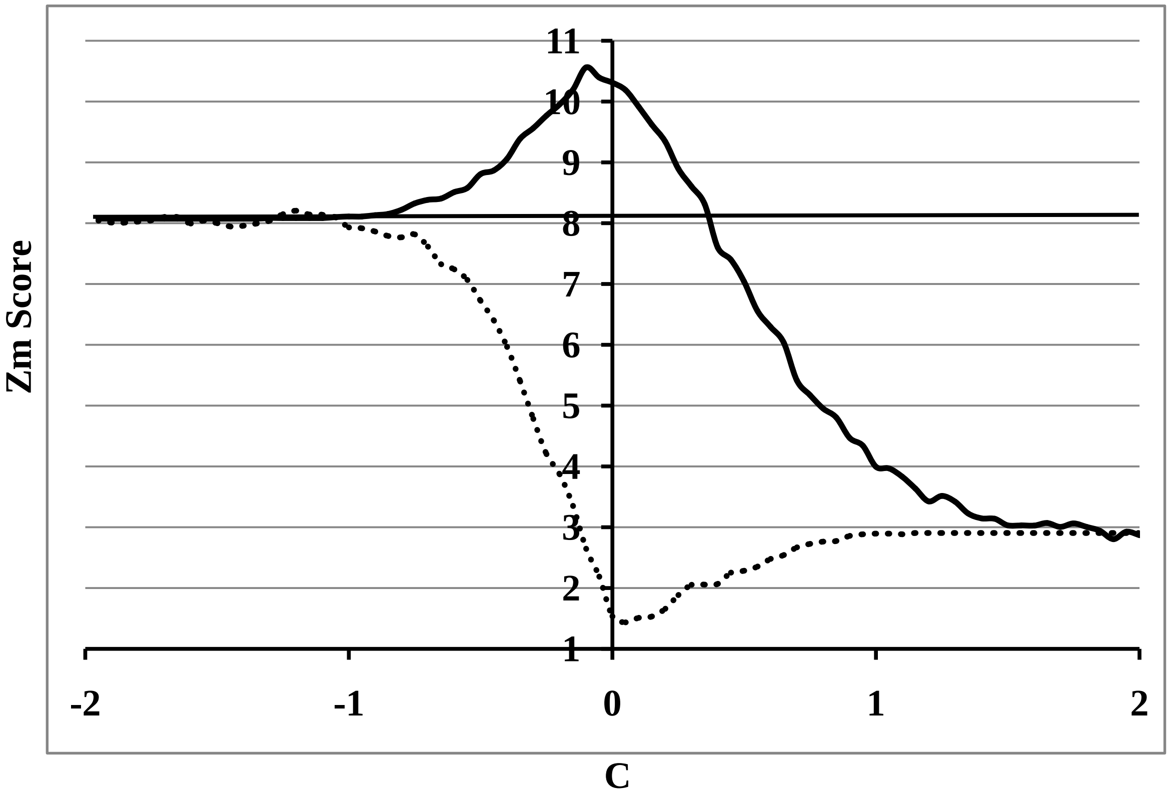

Figure5 Comparison of Zm-o, Zm-p, Zm-q values of CREB(Zm-o horizontal line, Zm-p solid line, Zm-q dashed line)

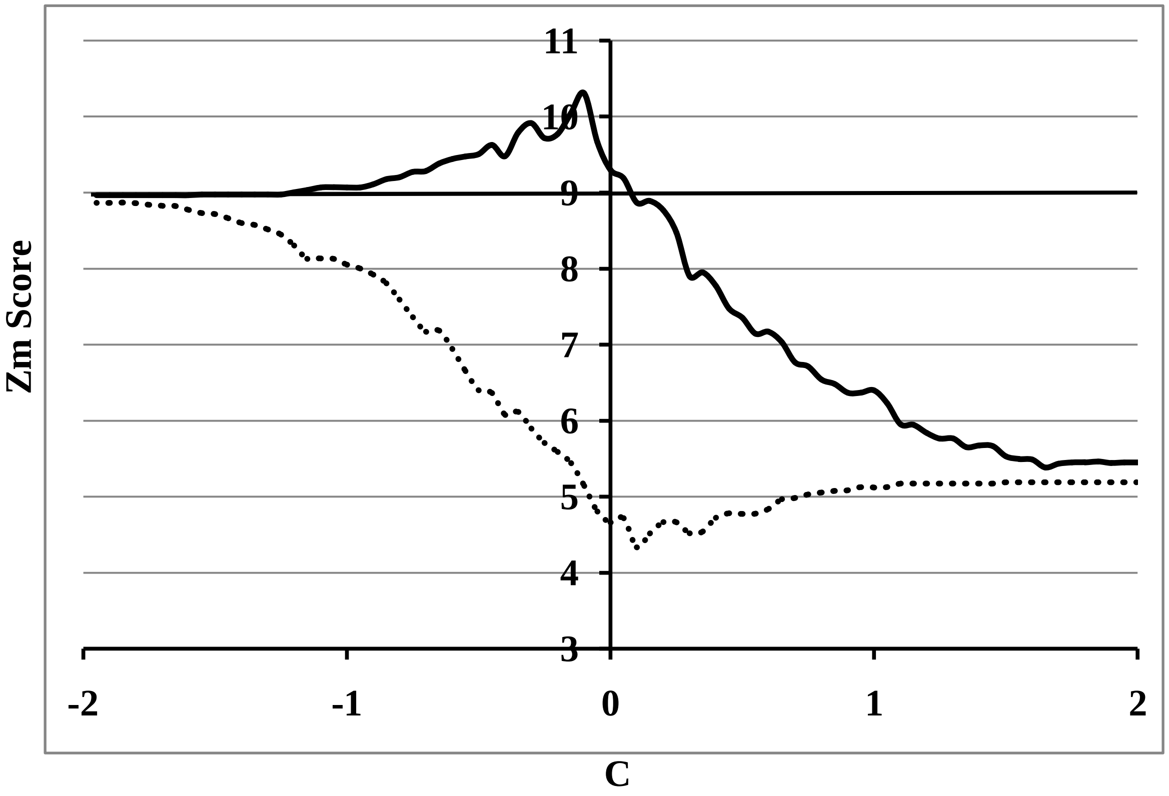

Figure6 Comparison of Zm-o, Zm-p, Zm-q values of Egr3(Zm-o horizontal line, Zm-p solid line, Zm-q dashed line)

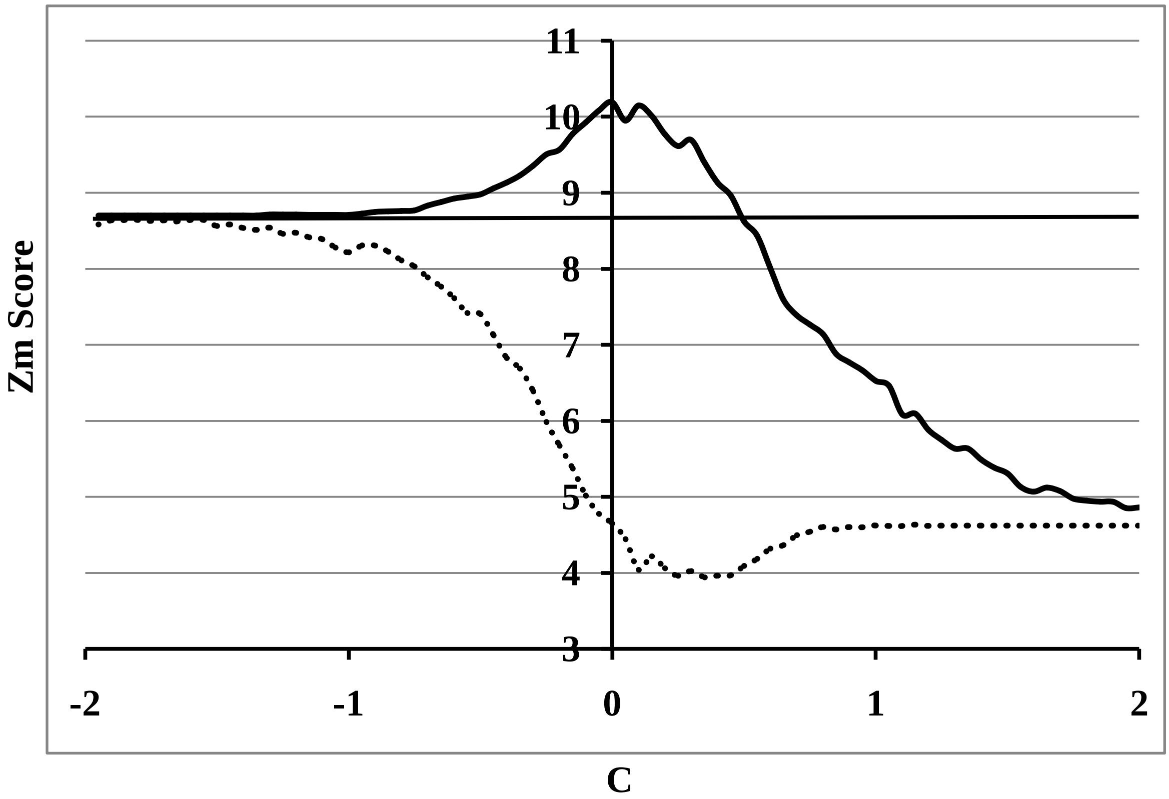

Figure7 Comparison of Zm-o, Zm-p, Zm-q values of KROX(Zm-o horizontal line, Zm-p solid line, Zm-q dashed line)

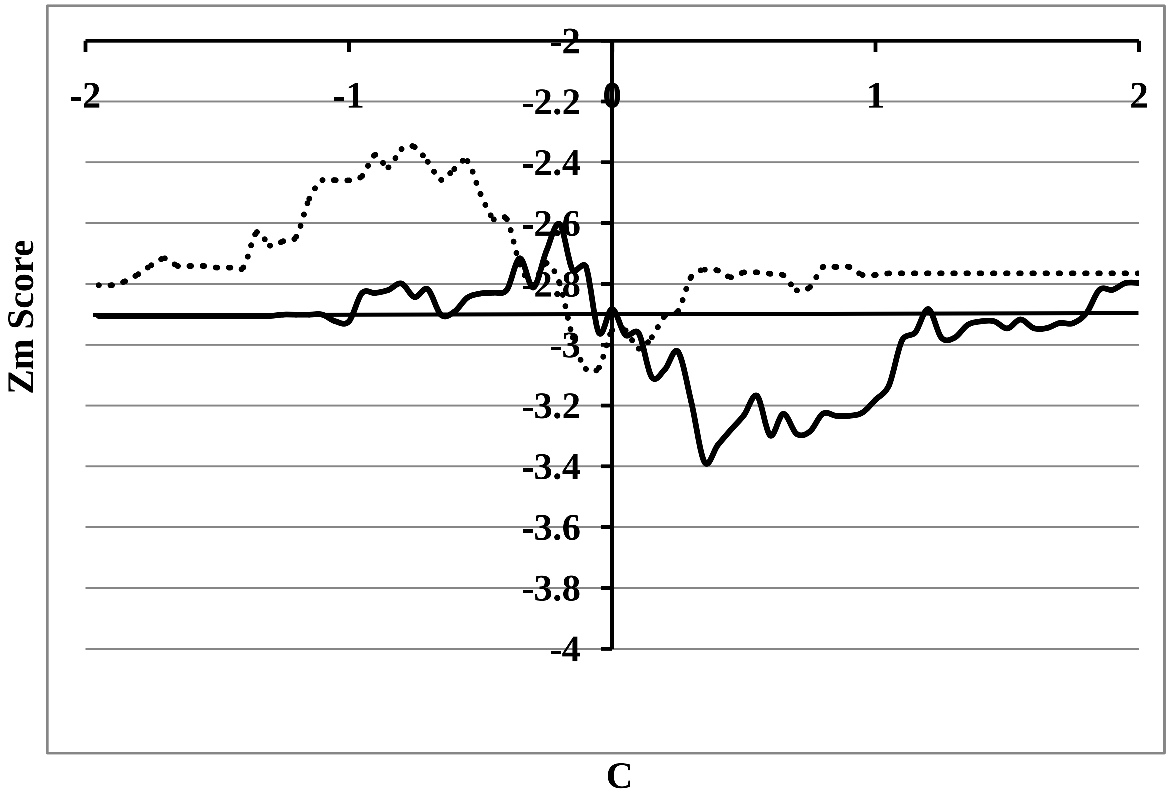

Figure8 Comparison of Zm-o, Zm-p, Zm-q values of GCNF(Zm-o horizontal line, Zm-p solid line, Zm-q dashed line)
